# CD8 T‐cell responses against the immunodominant *Theileria parva* peptide Tp2_49–59_ are composed of two distinct populations specific for overlapping 11‐mer and 10‐mer epitopes

**DOI:** 10.1111/imm.12637

**Published:** 2016-07-25

**Authors:** Timothy K. Connelley, Xiaoying Li, Niall MacHugh, Didier Colau, Simon P. Graham, Pierre van der Bruggen, Evans L. Taracha, Andy Gill, William Ivan Morrison

**Affiliations:** ^1^ Division of Immunity and InfectionThe Roslin InstituteThe University of EdinburghMidlothianUK; ^2^ Ludwig Institute for Cancer Research and de Duve InstituteUniversite catholique de LouvainBrusselsBelgium; ^3^ The International Livestock Research InstituteNairobiKenya; ^4^ Division of NeurobiologyThe Roslin InstituteThe University of EdinburghMidlothianUK; ^5^ Present address: School of Life Sciences and TechnologyXinxiang Medical UniversityLaboratory Building Room 232XinxiangHenanCN 453003China; ^6^ Present address: The Pirbright InstituteAsh RoadPirbrightGU24 0NFUK; ^7^ Present address: Institute of Primate ResearchPO Box 24481‐00502KarenKenya

**Keywords:** cattle, CD8 T cell, epitope, tetramer, *Theileria parva*

## Abstract

Immunity against *Theileria parva* is associated with CD8 T‐cell responses that exhibit immunodominance, focusing the response against limited numbers of epitopes. As candidates for inclusion in vaccines, characterization of responses against immunodominant epitopes is a key component in novel vaccine development. We have previously demonstrated that the Tp2_49–59_ and Tp1_214–224_ epitopes dominate CD8 T‐cell responses in BoLA‐A10 and BoLA‐18 MHC I homozygous animals, respectively. In this study, peptide–MHC I tetramers for these epitopes, and a subdominant BoLA‐A10‐restricted epitope (Tp2_98–106_), were generated to facilitate accurate and rapid enumeration of epitope‐specific CD8 T cells. During validation of these tetramers a substantial proportion of Tp2_49–59_‐reactive T cells failed to bind the tetramer, suggesting that this population was heterogeneous with respect to the recognized epitope. We demonstrate that Tp2_50–59_ represents a distinct epitope and that tetramers produced with Tp_50–59_ and Tp_49–59_ show no cross‐reactivity. The Tp2_49–59_ and Tp2_50–59_ epitopes use different serine residues as the N‐terminal anchor for binding to the presenting MHC I molecule. Molecular dynamic modelling predicts that the two peptide–MHC I complexes adopt structurally different conformations and Tcell receptor *β* sequence analysis showed that Tp2_49–59_ and Tp2_50–59_ are recognized by non‐overlapping T‐cell receptor repertoires. Together these data demonstrate that although differing by only a single residue, Tp2_49–59_ and Tp2_50–59_ epitopes form distinct ligands for T‐cell receptor recognition. Tetramer analysis of *T. parva*‐specific CD8 T‐cell lines confirmed the immunodominance of Tp1_214–224_ in BoLA‐A18 animals and showed in BoLA‐A10 animals that the Tp2_49–59_ epitope response was generally more dominant than the Tp2_50–59_ response and confirmed that the Tp2_98–106_ response was subdominant.

## Introduction

CD8 T cells play a key role in immunity against a range of intracellular pathogens. The antigenic specificity of individual T cells is determined by clonally distributed T‐cell receptors (TCR) that recognize short pathogen‐derived peptide fragments (generally 8–12 amino acids in length) presented in the context of the host's MHC class I (MHC I) molecules on the surface of infected cells. Although the proteome of most pathogens will contain a multitude of peptides that have the potential to act as MHC I‐associated epitopes, the CD8 T‐cell response tends to be focused on a limited number of dominant epitopes. Due to their inherent immunogenicity, such ‘immunodominant’ epitopes are considered to be good candidates for inclusion in subunit vaccines and, as such, identification and characterization of the immune responses against these epitopes is a critical component of vaccine development.

Mature MHC I proteins are hetero‐trimeric complexes, composed of a peptide bound to a highly polymorphic MHC I *α* heavy chain, non‐covalently associated with invariant *β*_2_‐microglobulin. *In vivo* assembly of the peptide–MHC I (pMHC I) is a complex and multi‐staged process that is tightly regulated by a number of chaperones. The repertoire of peptides that can bind to any MHC I is constrained, first by the dimensions of the peptide‐binding groove, which is closed at both ends, so limiting the size of the peptide fragments, and second by the presence of ‘pockets’ within the groove that preferentially accommodate certain amino acids at particular positions in the peptide. Sequence polymorphisms between MHC I molecules result in morphologically distinct ‘pockets’ and consequently variation of the repertoire of peptides that can bind stably. For each MHC I allele, the majority of peptides that have the capacity to bind share a ‘peptide motif’, which reflects preferential usage of certain amino acids in residues involved in ‘anchoring’ the peptide to the MHC I molecule.

East Coast Fever is an economically important disease of cattle caused by the tick‐borne protozoan parasite *Theileria parva*, which is prevalent in large areas of eastern and southern sub‐Saharan Africa. East Coast Fever is estimated to kill over 1 million animals per year and is a major socio‐economic constraint on the livelihoods of pastoralists in the affected regions. Although East Coast Fever is often lethal, cattle that recover naturally, or are immunized by an ‘infection and treatment method’ of vaccination, develop long‐lasting strain‐specific immunity.^1^ Immune animals exhibit potent CD8 T‐cell responses specific for lymphocytes infected with the schizont stage of the parasite and there is strong evidence that these CD8 T cells play a key role in immunity.^2^, ^3^, ^4^, ^5^, ^6^ The identification of a number of antigens and epitopes recognized by parasite‐specific CD8 T cells in animals of defined MHC I genotypes has facilitated studies of the fine antigenic specificity of these responses.^7^, ^8^ Using *in vitro* cytotoxicity assays to analyse large sets of CD8 T‐cell clones, we have demonstrated that two of these epitopes – Tp1_212–224_ and Tp2_49–59_ – are highly immunodominant, accounting for > 60% of the CD8 T‐cell response, in animals homozygous for BoLA‐A10 (A10) and BoLA‐A18 (A18) MHC I haplotypes, respectively.^9^

The advent of pMHC I tetramer reagents, composed of fluorescently labelled tetrameric complexes of recombinant MHC I proteins loaded with defined peptides,^10^ has provided a more rapid and precise method for identifying, quantifying and characterizing epitope‐specific T cells, which is independent of the functional properties of the cells. Herein, we describe studies using pMHC I tetramers to quantify the CD8 T cells specific for three *T. parva* epitopes in the Tp1 and Tp2 antigens – Tp1_212–224_, Tp2_49–59_ and Tp2_98–106_ – in both *in vitro* cultured CD8 T‐cell lines and *ex vivo* T‐cell populations. During these studies we demonstrate that the previously defined Tp2_49–59_‐specific T‐cell response in A10 animals is composed of two distinct populations specific for Tp2_49–59_ and Tp2_50–59_. These two epitopes use alternative N‐terminal serine residues (S_50_ and S_51_, respectively) to bind to the presenting MHC I molecule (2*1201); molecular dynamics simulations indicate that as a result of this the Tp2_49–59_ and Tp2_50–59_ peptides assume distinct conformations when bound to 2*1201, leading to differential TCR recognition and CD8 T cells with distinct specificities for either Tp2_49–59_ or Tp2_50–59_.

## Materials and methods

#### Animals, immunization and challenge

Holstein Friesian cattle homozygous for either the A10 or A18 MHC I haplotypes were selected for the study by a combination of serological typing with MHC I‐specific monoclonal antibodies^11^ and MHC I allele‐specific PCR.^12^ Cattle were immunized against the Muguga stock of *T. parva* by infection with cryopreserved sporozoites and simultaneous administration of a long‐acting formulation of oxytetracycline, as described previously.^1^ Some of the animals were challenged with a lethal dose of sporozoites at specified time periods after immunization. All experimental animal work was completed in accordance with the UK Animals (Scientific Procedures) Act 1986.

#### Generation of *T. parva*‐specific CD8 T‐cell lines and clones

*Theileria parva*‐specific CD8 T‐cell lines were generated as described previously.^13^ In brief, peripheral blood mononuclear cells (PBMC) harvested from immunized animals by density gradient centrifugation were stimulated three times at weekly intervals by co‐culture with *γ*‐irradiated (60 Gy) autologous *T. parva*‐infected cells. Before the third stimulation, cell lines were depleted of CD4 T cells and *γδ* T cells by complement‐mediated lysis with lineage‐specific antibodies (CD4: IL‐A12, IgG2a,^14^ and TCR‐*γδ*: GB21A, IgG2b, VMRD, Washington State University, WA). During the third stimulation, the culture medium was supplemented with 100 U/ml of recombinant human interleukin‐2 (IL‐2; Chiron, Emeryville, CA). Clones were generated from the CD8 T‐cell‐enriched cell lines by limiting dilution 7 days after the third stimulation and expanded by re‐stimulation as detailed elsewhere.^13^ All tissue culture was conducted in RPMI‐1640 medium supplemented with 10% (v/v) fetal bovine serum (FBS), 20 mm HEPES buffer, 50 μm *β*‐mercaptoethanol, 2 mm l‐glutamine, 100 U/ml penicillin and 100 μg/ml streptomycin.

Purified populations of CD8 T cells specific for Tp2_49–59_ and Tp2_50–59_ used for TCR‐*β* (TRB) chain sequence analysis were derived from CD8 T cells purified from PBMC of immunized animals by MACS separation (Miltentyi Biotec, Bisley, UK) using a CD8‐specific monoclonal antibody (IL‐A51–IgG1^15^), and cultured for 10–11 days in the presence of autologous *T. parva*‐infected cells. Epitope‐specific T cells were purified from these cultures by tetramer staining followed by cell sorting as described below and then re‐stimulated for an additional 7 days. Purity of tetramer‐sorted populations was verified as being > 98% before mRNA extraction.

#### Tetramer production

The cDNA clones of the BoLA class I alleles 2*01201 and 6*01301 were used as templates to amplify by PCR the sequence coding for the extracellular domain (amino acids G_22_‐P_307_) of the heavy chains. The PCR products were cloned into a derivative of plasmid pET3D (Novagen, Merck, Nottingham, UK) containing a BirA biotinylation site in‐frame with the 3′ end of the heavy chain sequences. The sequence coding for the mature bovine *β*_2_‐microglobulin (amino acids I_21_‐L_118_) was cloned into a derivative of pET9 (Novagen). The proteins were expressed in *Escherichia coli* BL21 (DE3) as inclusion bodies. The cell cultures were lysed by high‐pressure cell disruption, inclusion bodies collected by centrifugation and washed extensively with 50 mm Tris–HCl, 100 mm NaCl, 1 mm EDTA, 1 mm dithiothreitol, and 0·5% (w/v) sodium deoxycholate (pH 8). Washed inclusion bodies were solubilized, for the heavy chains, in 6 m guanidine hydrochloride, 100 mm Tris–HCl (pH 8), and for the *β*_2_‐microglobulin, in 8 m urea, 25 mm sodium acetate (pH 4).

Recombinant MHC I/peptide complexes were folded by direct dilution of the denatured heavy chain (1 μm), *β*_2_‐microglobulin (2 μm) and the peptide (10 μm) in the following refolding mixture: 50 mm Tris–HCl, 400 mm l‐arginine hydrochloride, 2 mm EDTA, 5 mm reduced glutathione, 0·5 mm oxidized glutathione and 0·1 mm PMSF (pH8·5). The mixture was incubated for 72 hr at 4° and then concentrated by ultrafiltration. The soluble MHC/peptide complexes were purified by gel filtration and biotinylated using the BirA enzyme (Avidity LCC, Denver, CO). Fluorescent multimers were obtained by mixing biotinylated MHC/peptide complexes with streptavidin–phycoerythrin conjugate (BD Pharmingen, Franklin Lakes, NJ).

#### Tetramer staining and purification of tetramer‐positive cells

Tetramer‐positive populations within PBMC were analysed using multi‐colour flow cytometry. In brief, PBMC (1 × 10^7^/ml) were stained with phycoerythrin‐labelled tetramers at a final concentration of 20 nm for 30 min at room temperature, washed twice in PBS containing 0·5% FBS before staining with a mixture of monoclonal antibodies against CD4 (IL‐A12), TCR‐*γδ* (GB21A), CD21 (CC21, IgG1^16^), NKp46 (EC1.1 IgG1^17^) and CD172a (IL‐A24, IgG1^18^) for 30 min at 4°. After three washes, cells were incubated for 30 min at 4° with Alexa Fluor 647conjugated goat anti‐mouse IgG (Life Technologies, Paisley, UK), washed three times, and then SYTOX Red Dead Cell Stain (Life Technologies) was added. Flow cytometric analysis was then conducted on a FACSCalibur or cell‐sorting on a FACSAria (both BD Biosciences, Oxford, UK). All monoclonal antibodies had been titrated to determine optimal dilutions before the experiment and the combination of antibodies and stains had been demonstrated to leave viable CD8 *αβ* T cells as the only unstained population (data not shown), which could therefore be gated as the Alexfluor647/SYTOX Red‐negative subset. For tetramer staining of cultured CD8 T cells staining with lineage‐specific antibodies was excluded.

#### Cytotoxicity and MHC I‐binding competition assays

Standard 4‐hr [^111^In]‐release cytotoxicity assays were used to examine the epitope‐specificity of CD8 T‐cell clones, using autologous *Theileria annulata*‐infected cells that had been incubated with peptides (100 ng/ml) for 1 hr before the assay as target cells. All assays were conducted in duplicate, and controls included *Theileria annulata*‐infected target cells without added peptide. Percentage specific‐lysis was calculated as: [(sample release – spontaneous release) × 100%/(maximal release – spontaneous release)] and expressed as the mean of the duplicated assays. Maximal and spontaneous release were derived from triplicates of target cells incubated with 0·2% (v/v) Tween‐20 and RPMI‐1640 medium containing 5% (v/v) FBS, respectively. All peptides in this study were supplied by Pepscan Systems (Lelystad, the Netherlands).

The capacity of variants of the Tp2_49–59_ and Tp2_50–59_ peptides to bind MHC I was determined by their ability to compete with Tp2_98–106_, another epitope also presented by the 2*01201 MHC I allele.^7^ Autologous *T. annulata*‐infected target cells were pre‐incubated with individual peptides at serial threefold dilutions ranging either from 3 μg/ml to 30 ng/ml or from 5 μg/ml to 10 ng/ml for 1 hr before the addition of Tp2_98–106_ at 100 ng/ml, so that the competitor/target peptide ratio ranged from either 30 : 1 to 0·3 : 1 or 50 : 1 to 0·1 : 1 respectively. After incubation for a further hour, 4 hr [^111^In]‐release cytotoxicity assays were performed using a Tp2_98–106_‐specific CD8 T‐cell clone at an effector to target ratio of 10 : 1. The MHC‐binding capacity of the Tp2_49–59_ and Tp2_50–59_ peptide variants was reflected in the inhibition of cytotoxicity as a consequence of competitive blocking of MHC binding and presentation of the Tp2_98–106_ peptide.

#### TRB chain sequencing

For sequencing of TRB chains expressed by CD8 T‐cell clones, total RNA was extracted using Tri‐reagent (Sigma‐Aldrich, Poole, UK) and cDNA synthesized using the Reverse Transcription System (Promega, Madison, WI) with priming by the Oligo (dT)_15_ primer, according to the manufacturer's instructions. TRB chains were PCR amplified using either V*β*‐subgroup‐specific or pan‐V*β* primers as described previously^19^, ^20^ and the products were sequenced. For sequencing of tetramer‐sorted polyclonal populations TRB chains were amplified using a SMART PCR protocol, modified from that described in Quigley *et al*.,^21^ that allows unbiased TRB chain sequencing. In brief, mRNA was extracted from tetramer‐sorted CD8 T cells using Oligotex Direct mRNA kit (Qiagen, Manchester, UK) and cDNA was synthesized with the SMARTer RACE cDNA Kit (Clontech, Paris, France). The cDNA was then cleaned using NucleoSpin Extract II (Clontech) before PCR amplification with a bovine TRB constant gene‐specific primer (5′‐GGAGATCTCTGCTTCCGAGGGTTC‐3′). PCR products were subjected to agarose gel electrophoresis and extracted, purified using NucleoSpin Extract II, ligated into pGEM‐T Easy (Promega) and sub‐cloned into JM109 *E. coli*. The inserts in individual colonies were PCR amplified and the products were sequenced. Sequence analysis was performed using the dnasis max v2.0 software package under default conditions (Miriabio, Alameda, CA). Throughout the manuscript, nomenclature based on the World Health Organization‐IUIS TCR nomenclature system as used by Arden *et al*.^22^ has been employed for V*β* genes, whereas the J*β* gene nomenclature is based upon their organization in the bovine TRB locus.^23^

#### Molecular dynamics simulations

The coordinates for the bovine MHC class I allele 6*01301 bound to both *β*_2_‐microglobulin and to the 11‐mer peptide from *T. parva*, Tp1_214–224_ were obtained (pdb code 2XFX^24^). By using the deepview Swiss PDB Viewer, the sequence of MHC I allele 2*1201 was threaded into the structure of the 6*01301 allele and the sequence of the Tp2_49–59_ peptide was threaded into the structure of the Tp1_214–224_ peptide; the *β*_2_‐microglobulin structure was left unchanged. The new model was energy minimized in deepview and coordinates were saved. A replicate model with the 10‐mer peptide Tp2_50–59_ was created by removing a central amino acid from the peptide structure, re‐ligating the peptide backbone, re‐creating the correct amino acid sequence and then energy minimizing in deepview. This process yielded two starting structures for simulations.

All molecular dynamics simulations were performed by use of the AMBER 7 suite of programmes and essentially followed previously published methods for estimating the structures of peptides bound to MHC molecules.^25^ In all simulations the Gibbs approximation of implicit water was used with a salt concentration of 0·1 m, a non‐bonded cut‐off of 12·0 Å and a step length of 1 fs. Briefly, structures were extensively energy minimized to remove local hotspots introduced by the threading/mutagenesis procedures and were then subjected to a simulated annealing process followed by constant temperature molecular dynamics at 283 K. During the annealing phase the temperature was raised to 1500 K over 80 ps, held at 1500 K for a further 80 ps and then reduced to 283 K over 800 ps before a final hold at 283 K for 40 ps (total annealing time 1 ns). During the initial energy minimization an energy constraint of 5 kcal/mol was applied to both the MHC I molecule and *β*_2_‐microglobulin, whereas during the simulated annealing these restraints were raised to 20 kcal/mol. Also, during the annealing phase, three distance constraints were applied to ensure that the peptide remained in the binding groove – these constraints limited hydrogen bond lengths between: (i) OG1 atom of Thr 148 of MHC I and the C‐terminal O atom of the peptide; (ii) OH atom of Tyr 164 of MHC I and the carbonyl oxygen of peptide residue 1; and (iii) the OH atom of Tyr 176 of MHC I and the N‐terminal N atom of peptide residue 1. In all simulations the ‘shake’ algorithm was applied to limit bond lengths involving hydrogen atoms. The final structures after simulated annealing were energy minimized without restraints and structures were subjected to 4 ns of molecular dynamics at 283 K in the presence of the above three distance constraints and with 20 kcal/mol positional restraints applied to the MHC I and *β*_2_‐microglobulin molecules. Coordinates were saved at regular intervals throughout the molecular dynamics simulations and analysed using VMD; VMD is developed with NIH support by the Theoretical and Computational Biophysics group at the Beckman Institute, University of Illinois at Urbana‐Champaign, USA.^26^

## Results

### A subset of Tp2_49–59_ peptide‐reactive CD8 T cells do not bind the Tp2_49–59_/2*01201 tetramer

Previous analyses of panels of cloned CD8 T‐cell lines demonstrated that the responses of A10 and A18 homozygous cattle against *T. parva* Muguga were dominated by T cells specific for the Tp2_49–59_ (KSSHGMGKVGK) and Tp1_214–224_ (VGYPKVKEEML) epitopes respectively.^9^ In these analyses Tp2_98–106_ (QSLVCVLMK) was found to be a subdominant epitope in homozygous A10 responses. As part of efforts to extend our studies to include *ex vivo* analyses, pMHC I tetrameric complexes were generated with the relevant MHC I heavy chains (6*01301 for Tp1_214–224_ and 2*01201 for both Tp2_49–59_ and Tp2_98–106_).

In initial experiments to validate these tetramers, epitope‐specific CD8 T‐cell clones and polyclonal T‐cell lines (from which defined percentages of epitope‐specific T‐cell clones had been determined by cytotoxicity assays against peptide‐loaded autologous targets), were stained with the tetramers (Table 1). The percentage of Tp1_214–224_‐tetramer‐positive cells in polyclonal *T. parva* Muguga‐specific CD8 T‐cell lines from two A18 homozygous animals corresponded well with results from clonal analysis. Similarly, the percentage of Tp2_98–106_‐tetramer‐reactive cells in polyclonal *T. parva* Muguga‐specific CD8 T‐cell lines from two A10 homozygous animals approximated to that determined by epitope‐specific cytotoxicity results. In contrast, the percentage of Tp2_49–59_‐tetramer‐positive cells in the A10 homozygous lines was considerably lower than that indicated from the clonal analyses of the lines. Examination of individual CD8 T‐cell clones (*n* = 29) demonstrated that half of those shown to react with the Tp2_49–59_ peptide did not stain with the Tp2_49–59_‐tetramer (Table 2). These data suggested that the Tp2_49–59_‐specific populations defined by cytotoxicity assays were in fact heterogeneous and included a component for which Tp2_49–59_ was not the epitope.

**Table 1:** Frequency of epitope‐specific populations in polyclonal *Theileria parva*‐specific CD8 T‐cell lines from BoLA‐A10 and BoLA‐18 homozygous animals identified by clonal analysis and peptide–MHC I‐tetramer staining

Animal	Immune status	BoLA‐A18		BoLA‐A10					
		Tp1_214–224_		Tp2_98–106_		Tp2_49–59_			
		Clonal analysis	Tetramer	Clonal analysis	Tetramer	Clonal analysis	Tp2_49–59_ Tetramer	Tp2_50–59_ Tetramer	Tp2_49–59__+_ Tp2_50–59_ Tetramer
592 (A10)	PI			3%	3·5%	60%	23·8%	20·0%	43·8%
	PC			10%	11·6%	80%	32·2%	32·5%	64·7%
1011 (A10)	PI			2%	3·1%	74%	56·0%	10·5%	66·5%
	PC			2%	1·0%	61%	40·9%	27·7%	68·6%
641 (A18)	PI	78%	70·0%						
468 (A18)	PI	81%	76·4%						

The percentage of epitope‐specific T cells in polyclonal *T. parva*‐specific CD8 T‐cell lines obtained from BoLA‐A10 (592 and 1011) and BoLA‐A18 homozygous animals (641 and 468) as defined by analysis of cytotoxic activity of panels of cloned CD8 T cells against peptide‐loaded autologous targets (clonal analysis) and direct pMHC I tetramer staining. Post‐immunization (PI) Post‐challenge (PC). IFNG analysis on subsets of clones indicated that the frequency of epitope‐specific (i.e. IFNG^+^) but non‐cytotoxic cells in the lines examined was negligible (data not shown) and therefore cytotoxicity has been taken as a proxy for epitope‐specificity.

**Table 2:** Peptide–MHC I tetramer staining of *Theileria parva*‐specific CD8 T‐cell clones derived from BoLA‐A10 homozygous animals that cytotoxic analysis had identified as Tp2_49–59_‐specific

Clone	TRB sequence			Tetramer staining	
	TRBV	CDR3	TRBJ	Tp2_49–59_	Tp2_50–59_
1011.1‐1	1.6	SQVGGIYGEL	3s1	−	+
592.54‐1	1.7	SPNSYEQ	3s7	−	+
592.56‐1	1.7	SPNSYEQ	3s7	−	+
592.77‐1	2.4	QWGGSYEEQ	2S1	−	+
1011.37‐1	2.4	RFGPGGLSYEQ	3S7	−	+
592.15‐1	3.1	SRKGGGLQSTQ	2S3	+	−
592.43‐1	10.2	SQADSGAYEQ	3S7	+	−
592.43‐3	12.1	HIRGGLDTQPL	3S2	−	+
1011.68‐3	12.2	SYSPGGGSPL	3S3	+	−
1011.12‐1	12.2	SYSPGGGSPL	3S3	+	−
592.9‐1	13.2	SHAGYEQ	3S7	+	−
1011.13‐1	14.1	SVGNSNYEQ	3s7	+	−
1011.21‐1	14.1	SVGNSNYEQ	3s7	+	−
1011.25‐1	14.1	SVGNSNYEQ	3s7	+	−
1011.16‐3	14.1	SVGNSNYEQ	3s7	+	−
1011.22‐3	14.1	SVGNSNYEQ	3s7	+	−
1011.21‐3	14.1	SVGNSNYEQ	3s7	+	−
1011.29‐3	14.1	SVGNSNYEQ	3s7	+	−
1011.31‐3	14.1	SVGNSNYEQ	3s7	+	−
1011.6‐3	28.1	AEYGGENTQPL	3s2	−	+
1011.15‐3	28.1	AEYGGENTQPL	3s2	−	+
1011.35‐3	28.1	AEYGGENTQPL	3s2	−	+
592.35‐1	28.1	AEYGGENTQPL	3s2	−	+
592.50‐1	28.1	AEYGGENTQPL	3s2	−	+
592.59‐1	28.1	AEYGGENTQPL	3s2	−	+
592.18‐1	28.1	AEYGGENTQPL	3s2	−	+
592.26‐1	28.1	AEYGGENTQPL	3s2	−	+
592.55‐1	28.1	GGRDSIYDY	3s2	−	+
1011.5‐1	X	SKAAAEDGYEQ	3S7	+	−

For each clone the designated name (animal number, clone number and a ‘–1’ and ‘−3’ suffix referring to clones derived from lines established post‐immunization and post‐challenge respectively), sequence of the expressed TRB chains (composed of the TRBV gene, amino acid sequence of the CDR3 region and the TRBJ gene) and the result of staining with the Tp2_49–59_ and Tp2_50–59_ pMHC I tetramers are shown.

### Tp2_50–59_ is a novel 2*01201‐restricted *T. parva* epitope

**Figure 1:**
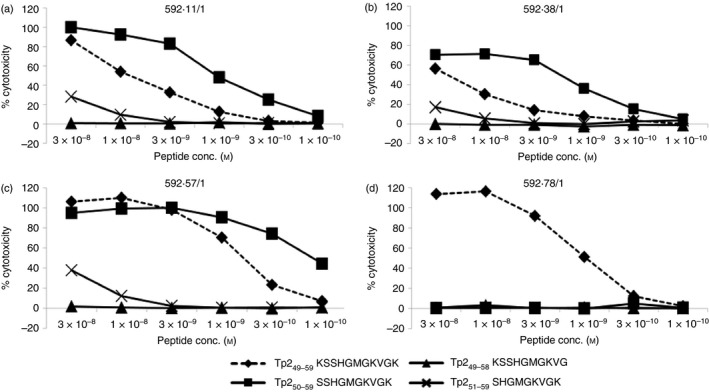
Minimal length peptide screening identifies Tp2_50–59_ as a novel epitope. Cytotoxic activity of Tp_49–59_‐lytic clones against titrated quantities of Tp_49–59_ and truncations of this peptide. Three clones – 592.11/1 (a), 592.38/1 (b) and 592.57/1 (c) – which lysed Tp_49–59_‐loaded autologous targets but did not stain with the Tp_49–59_‐tetramer all efficiently lysed Tp_50–59_‐ but not Tp_51–59_ or Tp_49–58_‐loaded targets. 592.78/1 (d) which stained with the Tp_49–59_ tetramer lysed Tp_49–59_‐loaded autologous targets but not targets loaded with any of the truncated peptides.

A potential reason for some Tp2_49–59_ peptide‐reactive T‐cell clones failing to bind the Tp2_49–59_‐tetramer is that they recognize a truncated version of the Tp2_49–59_ peptide. This could have been missed in the initial minimal epitope mapping of the Tp2_49–59_ epitope^7^ if the polyclonal CD8 T‐cell line used had a low frequency of T cells specific for a shorter epitope. We therefore performed minimal‐length peptide screening with synthetic peptides using three CD8 T‐cell clones that recognized Tp2_49–59_‐pulsed targets but failed to react with the Tp2_49–59_‐tetramer (Fig. 1). For all three clones the N‐terminal truncated 10‐mer Tp2_50–59_ (SSHGMGKVGK) was found to be the preferred epitope (Fig. 1a–c), maintaining the ability to elicit cytotoxicity at approximately a log lower peptide concentration (EC_50_ = 1 × 10^−9^ to 1 × 10^−10^ m) than the Tp2_49–59_ 11‐mer (EC_50_ = 1 × 10^−8^ to 1 × 10^−9^ m). Recognition of the 9‐mer peptide Tp2_51–59_ was substantially weaker, failing to elicit cytotoxicity at concentrations below 3 × 10^−8^ m. Analysis of a Tp2_49–59_‐tetramer‐reactive clone demonstrated that, as anticipated, lysis was observed only with cells incubated with the Tp2_49–59_ 11‐mer (Fig. 1d). Removal of one residue at the C‐terminus (Tp2_49–58_ 10‐mer) resulted in loss of T‐cell recognition by all four clones examined (Fig. 1a–d).

### Tp2_49–59_‐ and Tp2_50–59_‐specific CD8 T cells are distinct populations

**Figure 2:**
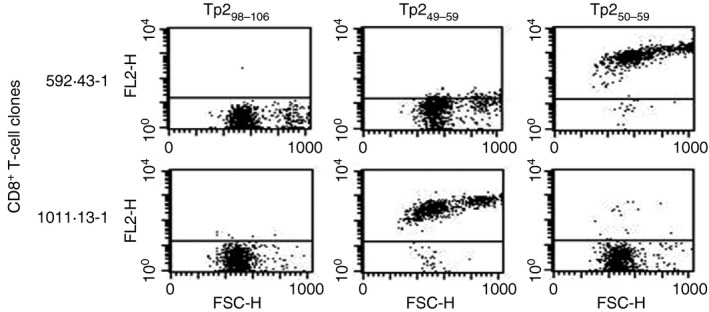
Tetramer staining of Tp2_49–59_‐ and Tp2_50–59_‐specific clones. Tetramer staining of representative Tp2_49–59_‐ (1011.13‐1) and Tp2_50–59_‐ (592.43‐1) specific clones demonstrates specificity of staining, with an absence of cross‐reactivity.

Based on these data, a Tp2_50–59_/2*01201 tetramer was generated and shown to positively stain all CD8 T‐cell clones that recognized Tp2_49–59_‐pulsed cells but failed to react with the Tp2_49–59_‐tetramer (Table 2). Conversely, those clones that stained with the Tp2_49–59_‐tetramer did not stain with the Tp2_50–59_‐tetramer, demonstrating that Tp2_49–59_‐ and Tp2_50–59_‐specificity is mutually exclusive (e.g. Fig. 2) Furthermore they indicated that the Tp2_49–59_‐ and Tp2_50–59_‐tetramer staining populations together constitute the total of the previously defined Tp2_49–59_‐specific populations; within the polyclonal lines the combined frequency of the Tp2_49–59_‐ and Tp2_50–59_‐tetramer stained cells were similar to that of Tp_49–59_‐specific cells obtained from cytotoxicity analysis (Table 1).

To further investigate the distinct nature of these populations, the TCR repertoires of sets of Tp2_49–59_‐ and Tp2_50–59_‐specific CD8 T cells were examined by sequencing of the expressed TRB chains. The CD8 T‐cell clones derived from the two A10 animals (592 and 1011) exhibited a broad sequence repertoire although there were dominant clonotypes present in both animals (Table 2). The TRB repertoires of the Tp2_49–59_‐ and Tp2_50–59_‐specific populations were largely ‘private’ (i.e. not shared between animals); however, there was one TRB sequence (VB28.1‐AEYGGENTQPL‐JB3s2) which was shared by a proportion of the Tp2_50–59_‐specific clones of the two animals. In contrast, there was no evident commonality of TRB sequences between the Tp2_49–59_‐ and Tp2_50–59_‐specific populations within the individual animals.

**Figure 3:**
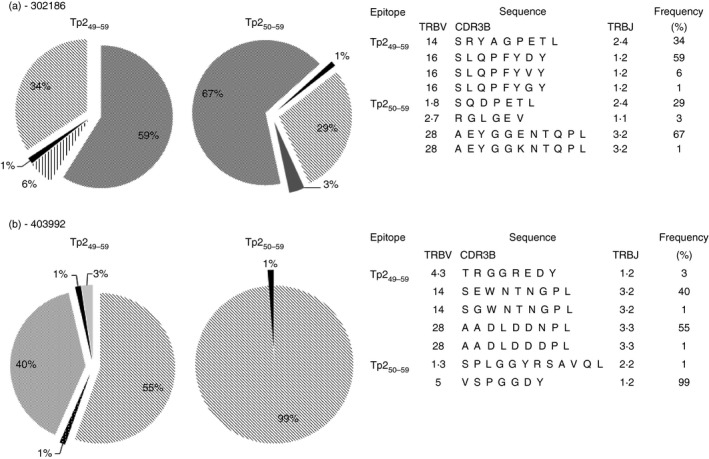
T‐cell receptor‐*β* (TRB) chain sequencing of Tp2_49–59_ and Tp2_50–59_‐sorted populations. TRB chains were sequenced from Tp2_49–59_ and Tp2_50–59_‐tetramer sorted populations from 2 BoLA‐A10 homozygous animals – (a) 302186 and (b) 403992 – using an unbiased SMART‐PCR protocol. The number of sequences generated from each population was > 70.

To provide a higher resolution analysis, larger numbers (> 70) of TRB chains were sequenced from Tp2_49–59_‐ and Tp2_50–59_‐tetramer‐sorted cell lines established from two additional A10‐homozygous animals (302186 and 403992), using a SMART‐PCR protocol that allows unbiased TRB chain sequencing in multi‐clonal populations. The Tp2_49–59_‐ and Tp2_50–59_‐specific populations were oligoclonal, being dominated by one or two large clonotypes, and the TRB repertoires were largely ‘private’ (Fig. 3), although the VB28.1‐AEYGGENTQPL‐JB3s2 TRB chain observed in animals 592 and 1011 was also identified in animal 302186. Critically, across the four animals no TRB sequence was shared between Tp2_49–59_‐ and Tp2_50–59_‐specific T cells. The disparity between the TRB chain repertoires of CD8 T cells specific for the two epitopes, indicate that they represent biophysically discrete pMHC I ligands for TCR recognition.

### Tp2_49–59_ and Tp2_50–59_ epitopes use different N‐terminal MHC I binding anchor residues

**Figure 4:**
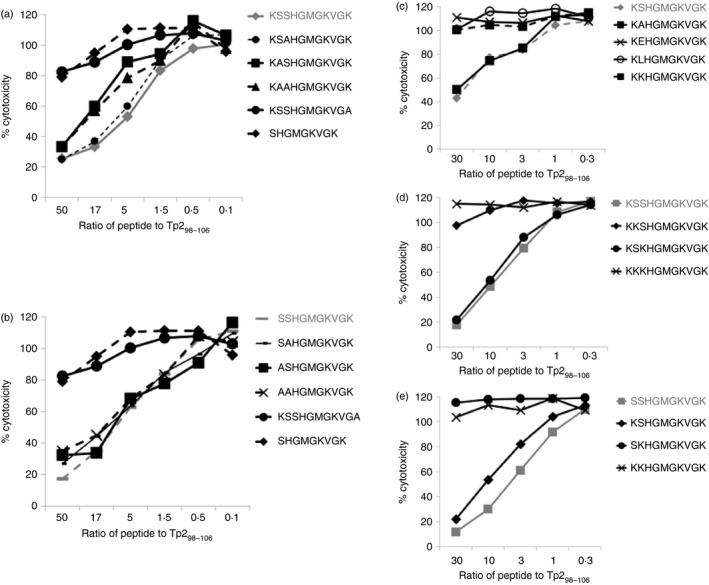
Tp2_49–59_ and Tp2_50–59_ bind to 2*01201 using different N‐terminal anchors. (a and b) An MHC I‐binding competition assay in which all permutations of serine and alanine at Tp2_50_ and Tp2_51_ were analysed demonstrates that the serine to alanine mutation does not abrogate MHC I binding of either the (a) Tp2_49–59_ or (b) Tp2_50–59_ epitopes, whereas lysine to alanine substitution of Tp2_59_ or removal of S_50_ does. (c) Mutant 10‐mer peptides in which S_50_ and S_51_ are replaced with single alanine (A), serine (S), glutamic acid (E), leucine (L) or lysine (K) residues demonstrate that the E, L and K substitutions all abrogate MHC I binding. MHC I competition assays in which all permutations of serine and lysine at Tp2_50_ and Tp2_51_ were analysed demonstrate that S_50_ and S_51_ are the N‐terminal anchor residues of Tp2_49–59_ (d) and Tp2_50–59_ (e) respectively. In each panel the index peptide (i.e. unmodified or minimally modified) sequence and data are shown in grey.

In a previous study, alanine scanning mutagenesis demonstrated that Lys_59_ was the C‐terminus anchor residue of the Tp2_49–59_ epitope but an N‐terminal anchor residue was not identified.^27^ MHccluster 2.0 predictions indicating that a serine (Ser), alanine (Ala) or threonine (Thr) at P2 is the preferred N‐anchor residue for 2*01201^28^ suggests that single‐alanine mutagenesis may not have affected MHC I binding either as a consequence of the similar biophysical characteristics of Ser and Ala (small and neutral) and/or the capacity of the tandem Ser_50_ and Ser_51_ residues to serve as alternative anchor residues. Further MHC I‐binding competition assays using peptides in which either or both Ser_50_ and Ser_51_ residues of both the Tp2_49–59_ (Fig. 4a) and Tp2_50–59_ (Fig. 4b) peptides were substituted with Ala demonstrated that all permutations of Ser and Ala at these positions maintained equivalent capacity to bind 2*01201, indicating that Ser to Ala substitution was insufficient to disrupt MHC I binding.

We therefore generated a series of mutant peptides in which the Ser_50_ and Ser_51_ residues were replaced with glutamic acid (Glu – large and negatively charged), leucine (Leu – large and neutral) and lysine (Lys – large and positively charged) residues at P2 and evaluated their performance, along with the peptides containing either a P2 serine or alanine substitution, in the MHC I‐binding competition assay (Fig. 4c). Consistent with our previous observations, the peptides with the Ser or Ala at P2 were able to bind to 2*01201, but the mutant peptides with Glu, Leu or Lys residues at P2 all exhibited no or only very limited binding to 2*01201. Based on these data, we repeated the MHC I‐binding competition assay with positions 50 and 51 of Tp2_49–59_ and Tp2_50–59_ substituted with all the different permutations of Ser and Lys. For both Tp2_49–59_ (Fig. 4d) and Tp2_50–59_ (Fig. 4e) the double lysine substitutions at positions 50 and 51 (Lys_50_Lys_51_) abrogated MHC I binding, as anticipated. For the Tp2_49–59_ peptide, the Lys_50_Ser_51_ but not the Ser_50_Lys_51_ substitution abrogated MHC I binding, whereas the reciprocal was true for Tp2_50–59_. These results indicate that Ser_50_ and Ser_51_ serve as the N′ terminal anchors for Tp2_49–59_ and Tp2_50–59_, respectively. A corollary of this is that for Tp2_49–59_ an extra amino acid has to be accommodated within the 2*01201 binding groove when compared with Tp2_50–59_, suggesting that the former must adopt a more ‘bulged’ conformation.

### Molecular dynamics simulations of Tp2_49–59_ and Tp2_50–59_ MHC I complexes predicts different peptide presentation to TCR

**Figure 5:**
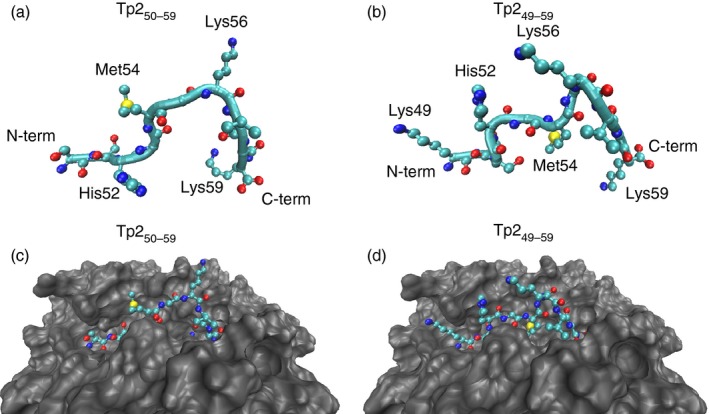
Modelling of the Tp2_49–59_ and Tp2_50–59_‐2*01201 structures. The conformation of Tp2_50–59_ (a) and (c) and Tp2_49–59_ (b) and (d) after simulated annealing. (a) and (b) show the peptide structures in isolation, whereas (c) and (d) show the same peptide structures *in situ* in the peptide binding groove of 2*01201. In each case, the peptide backbone is displayed as a continuous spline, whereas side chains are displayed as ball and stick representations. The MHC chain is displayed as a space‐filled representation. Figures were prepared using VMD.

To model the structural differences between the pMHC I complexes formed by Tp2_49–59_ and Tp2_50–59_ with 2*01201 at the atomic level we adopted a simulated annealing approach. The predicted structures of the pMHC complexes at the end of the simulated annealing procedure are shown in Fig. 5. As predicted, when bound to 2*01201 Tp2_49–59_ has a more ‘bulged’ conformation than Tp2_50–59_, with a greater component of the peptide protruding above the surface of the binding groove. However, more striking is the overall structural disparity between the two pMHC I complexes. First, at the N‐terminus the side chains of both Lys_49_ and His_52_ form prominent features in the Tp2_49–59_ complex that could form potential positively‐charged recognition sites for TCR. In contrast, in the model of Tp2_50–59_, Lys_49_ is obviously absent and the side‐chain of His_52_ is solvent‐protected, largely because the side chain of residue Arg_160_ of the MHC I chain, located on the lip of the binding groove, overlaps the His_52_ side‐chain. Second, although the side chains of Met_54_ and Lys_56_ in the central region of the pMHC I structures are surface‐exposed in both Tp2_49–59_ and Tp2_50–59_ and likely to be part of the pMHC I topography recognized by cognate TCR, the orientation of these two residues is markedly different. We performed further constant‐temperature molecular dynamics on these structures over 4 ns and analysed peptide conformations over 1 ps. The differences between the complexes were maintained throughout the simulations, demonstrating that the conformations depicted in Fig. 5 are representative of the entire data set. Hence, the two pMHC I complexes are predicted to present different structural motifs to the TCR, providing the mechanistic basis for why Tp2_49–59_ and Tp2_50–59_ are recognized by distinct TCR repertoires and consequently discrete CD8 T‐cell populations.

### The kinetics and relative immunodominance of Tp2 epitope‐specific responses *ex vivo*

**Figure 6:**
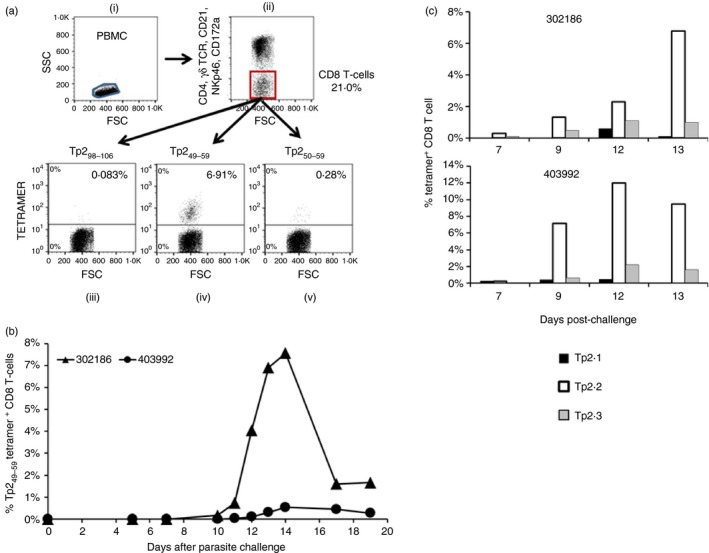
Use of tetramers to monitor the Tp2_49–59_, Tp2_50–59_ and Tp2_98–106_ responses in *Theileria parva*‐immunized BoLA‐A10 homozygous animals. (a) The gating strategy used to monitor the Tp2_49–59_, Tp2_50–59_ and Tp2_98–106_‐specific populations *ex vivo*. (b) *Ex vivo* frequency of tetramer‐specific CD8 T‐cell populations following an *in vivo* challenge with *T. parva* Muguga sporozoite stabilate and (c) following a single *in vitro* stimulation with autologous *T. parva* Muguga infected cell lines. (a) For gating (i) peripheral blood mononuclear cells (PBMC) were gated based on FSC/SSC parameters, (ii) *αβ* CD8 T cells were identified as being negative for the expression of CD4, TCR‐*γδ*, CD21, NKp46 and CD172a and (iii–v) epitope‐specific responses were identified by tetramer staining (b) Tp2_49–59_‐tetramer staining identified epitope‐specific populations in both 302186 and 403992. A Tp2_50–59_‐tetramer positive population was identified in 302186 (data not shown) but not 403992 whereas in neither animal was a Tp2_98–106_‐tetramer positive population identified. (c) Tetramer staining of CD8 T cells from 302186 and 403992 following a single *in vitro* stimulation with autologous *T. parva*‐infected cells identified Tp2_49–59_ (white bars), Tp2_50–59_ (grey bars) and Tp2_98–106_ (black bars) responses in both animals.

A primary purpose of developing the tetramers was to facilitate analysis and quantification of epitope‐specific CD8 T‐cell responses without the need for sustained *in vitro* culture and clonal analysis. Two A10 homozygous animals (403992 and 302186), previously immunized by the ‘infection and treatment method’ vaccination protocol, were challenged with a lethal dose of *T. parva* sporozoites and the CD8 T‐cell responses against Tp2_49–59_, Tp2_50–59_ and Tp2_98–106_ were monitored directly *ex vivo* using tetramers (Fig. 6a,b). In both animals a Tp2_49–59_‐specific response was evident, peaking at day 13/14 post‐challenge at 7·5% and 0·5% of CD8 T cells in 302186 and 403992, respectively. A response against Tp2_50–59_ was also observed in 302186, with a maximum of 0·28% of tetramer‐positive CD8 T cells on day 13 post‐challenge (data not shown). No Tp2_50–59_ response was detected in 403992 and no Tp2_98–106_‐tetramer‐positive population was seen in either animal.

To determine if the relative frequency of tetramer‐specific CD8 T cells observed following *in vitro* stimulation reflects that observed directly *ex vivo*, purified CD8 T‐cell cultures were established from these two animals by co‐culture with autologous *T. parva*‐infected cells and analysed over 13 days (Fig. 6c). Although the enrichment resulting from the *in vitro* stimulation enabled the detection of the subdominant Tp2_50–59_ and Tp2_98–106_ responses in these cultures, the dominance hierarchy of the Tp2_49–59_‐specific responses corresponded with the results obtained *ex vivo* during challenge.

## Discussion

Although pMHC I tetramers are widely used in humans and mice to analyse CD8 T‐cell responses, there is only one report of their use to examine responses in cattle.^29^ As part of studies to analyse the antigenic specificity of bovine CD8 T‐cell responses to *T. parva*, the present study set out to use pMHC I tetramers to quantify CD8 T‐cell responses to defined epitopes presented by two MHC I alleles. In addition to providing data on the relative abundance of T‐cell specificities, both *in vivo* and *in vitro*, the studies revealed that the response to one of the epitopes comprised two distinct CD8 T‐cell populations specific for 10‐mer and 11‐mer peptides within the same 11 amino acid sequence.

All three tetramers examined in the initial experiments stained discrete populations of cells in polyclonal CD8 T‐cell lines, in which the proportion of epitope‐specific T cells had been estimated previously by clonal analysis based on detection of cytolysis of peptide‐loaded target cells.^9^ The percentages of positive cells detected by two of the tetramers, Tp1_214–224_/6*01301 and Tp2_98–106_/2*01201, were consistent with the results obtained using cytotoxicity assays. In contrast, the proportion of Tp2_49–59_/2*01201 tetramer‐positive cells was substantially lower than expected based on the clonal analysis of the CD8 T‐cell lines and a number of clones shown to recognize the Tp2_49–59_ peptide failed to stain with the tetramer. Based on these observations, we tested the ability of three CD8 T‐cell clones, which reacted with the Tp2_49–59_ peptide but were not stained by the tetramer, to recognize a series of truncated peptides. The results of these experiments clearly identified Tp2_50–59_ as the minimal epitope for all three clones. We subsequently showed that all of the Tp2_49–59_‐reactive but Tp2_49–59_/2*01201 tetramer‐negative clones examined stained with a Tp2_50–59_/2*01201 tetramer, demonstrating that Tp2_49–59_ peptide‐reactive CD8 T‐cell populations were composed of two distinct subpopulations specific for overlapping epitopes of different lengths. The failure to identify the Tp2_50–59_ epitope in the initial epitope mapping studies^7^ probably reflects the absence or low frequency of Tp2_50–59_‐specific T cells in the polyclonal CD8 T‐cell line used in the analyses. Recently, another of the identified Tp2 epitopes, restricted by a different MHC allele, has been shown to be smaller than initially described.^29^ Together these data illustrate how tetramers can be used to verify and refine the epitopes identified by traditional epitope mapping techniques.

Few models analogous to the Tp2_49–59_/Tp2_50–59_ overlapping epitope scenario have been documented. One example that has been studied in detail is the HLA‐A2‐restricted response against the human melanoma antigen Melan‐A^MART‐1^, where both a nonamer (Melan‐A_27–35_ – AAGIGILTV) and decamer (Melan‐A_26–35_ – EAAGIGILTV) are CD8 T‐cell epitopes. Crystallographic studies have shown that the Melan‐A_27–35_ and Melan‐A_26–35_ epitopes use A_28_ and A_27_ residues as the N‐terminal anchors^30^ for MHC I binding respectively – the use of alternative tandem residues at the N‐terminus directly imitating the Tp2_49–59_/Tp2_50–59_ scenario. Similarly, the adoption of strikingly different conformations by Melan‐A_27–35_ and Melan‐A_26–35_ when bound to HLA‐A2 is emulated in the modelling results we present here for Tp2_49–59_ and Tp2_50–59_. Despite these similarities, there is a marked difference between the Melan‐A_27–35_/Melan‐A_26–35_ and Tp2_49–59_/Tp2_50–59_ systems with regard to tetramer binding. In the Melan‐A_27–35_/Melan‐A_26–35_ system, in addition to CD8 T‐cell subsets specific for either Melan‐A_27–35_ or Melan‐A_26–35_ there is also a subset that binds tetramers generated with both peptides.^31^ Detailed crystallographic analysis of the ternary TCR–pMHC structures formed by two cross‐reactive TCR (DMF4 and DMF5) with both Melan‐A_27–35_/HLA‐A2 and Melan‐A_26–35_/HLA‐A2 have provided insights into the molecular basis of this cross‐reactive capacity of Melan‐A‐specific T cells.^32^ Although DMF4 and DMF5 use different mechanisms to achieve cross‐recognition, for both TCRs, a critical common feature is that upon TCR ligation the nonameric peptide is lifted out of the MHC I groove and adopts a ‘bulged’ conformation that is more similar to that of the decamer. This ‘induced molecular mimicry’ has been described in a variety of pMHC I/TCR structures,^33^, ^34^ and in the case of Melan‐A_27–35_ the elevation of the peptide may be permitted by the sub‐optimal binding of the A_28_ residue with the N‐terminal pocket of the HLA‐A2 (which has a preference for a large residue such as leucine). In contrast, our data indicate that the Tp2_49–59_‐ and Tp2_50–59_‐tetramers are not cross‐reactively bound by T cells, suggesting that they effectively constitute two separate pMHC I entities, which are recognized by distinct repertoires of TCR. TRB chain sequence analysis confirmed that Tp2_49–59_‐ and Tp2_50–59_‐specific CD8 T cells use distinct, non‐overlapping TCR repertoires. Molecular modelling demonstrated that the differences between the Tp2_50–59_/2*01201 and Tp2_49–59_/2*01201 structures spanned much of the pMHC I‐surface with prominent N‐terminal features in Tp2_49–59_ formed by Lys_49_ and His_52_ being absent from Tp2_50–59_, and different orientations of the protruding Lys_56_ residue side chain towards the C‐terminus. Consequently there is limited commonality between the pMHC I structures available for binding by cognate TCRs and unlike the Melan‐A^MART‐1^ system, Tp2_49–59_ and Tp2_50–59_ use optimal N‐terminal (and C‐terminal) anchors, which may limit the capacity of TCR binding to alter the conformation of the pMHC I structures and so reduce the capacity for homogenization by ‘induced molecular’ mechanisms.

The Tp2 antigen is highly polymorphic, showing amino acid substitutions in 74% of the amino acid residues among 41 alleles that have been identified to date.^35^ We have shown that CD8 T cells directed against the Tp2_49–59_ and Tp2_50–59_ epitopes are parasite‐strain‐restricted and have proposed that the highly dominant nature of responses to these and other polymorphic epitopes is a major factor in the failure of different parasite strains to cross‐protect. The ability to generate two distinct pMHC I ligands from the same peptide sequence for T‐cell recognition, by eliciting separate CD8 T‐cell responses, has the potential to produce greater TCR diversity than either ligand alone, a feature that has been proposed to have benefits to host immunity by increasing the likelihood of cross‐reactivity between mutational epitope variants.^36^, ^37^, ^38^, ^39^ However, retrospective analysis of our previously published data^27^ demonstrates that Lys_56_ is critical for T‐cell recognition of both the Tp2_49–59_ and Tp2_50–59_ epitopes and it is notable that this residue is the most frequently substituted in natural allelic variants of the epitopes.^27^, ^35^ The protrusion of the Lys_56_ residue side chain above the pMHC I surface in models of both Tp2_49–59_/2*01201 and Tp2_50–59_/2*01201 suggests that regardless of their structural differences this is a critical TCR contact residue for both epitopes. Consequently, although this diversification strategy may confer some benefit with regard to greater probability of eliciting higher avidity T cells, the abrogation of both epitope‐specific responses through a single substitution (of residue Lys_56_) suggests the benefit to the host immune response may be limited. Instead, the focusing of two CD8 T‐cell responses on a single, highly polymorphic peptide,^27^ both of which are abrogated by the same single amino acid substitution, could benefit the pathogen by augmenting the strain‐restricted nature of the T‐cell response.

An initial objective in generating tetramers was to facilitate the tracking of epitope‐specific CD8 T‐cell responses to *T. parva in vivo*. Tetramer‐positive CD8 T cells were detected for the most dominant epitopes following parasite challenge of two previously immunized A10‐homozygous animals; cells positive for Tp2_49–59_ were first detected 9 days after challenge and reached peak levels (up to 7·5% of the CD8 T cells) at 12–13 days. The magnitude of these responses is similar to that reported recently by Svitek *et al*.,^29^ who used tetramers to monitor the Tp1_214–224_‐specific responses in A18+ animals following initial immunization with *T. parva*. The failure to detect tetramer‐positive cells *ex vivo* for the less dominant epitopes in our study reflects limitations in the sensitivity of the one‐step staining method we employed, which requires further refinement^40^ to improve sensitivity. The kinetics of the responses to the dominant epitopes following parasite challenge are similar to that observed in previous studies using limiting dilution analyses to monitor CD8 T‐cell responses to parasitized cells,^39^ although the latter assay detected a much lower frequency of responding cells than tetramers. It is of note that Taracha *et al*.^41^ observed that the peak of the response detected by measuring cytototoxicity occurs approximately 2 days earlier compared with detection by limiting dilution analyses, probably reflecting changes in the proportion of the specific cells that express effector function over time. These observations are consistent with evidence that tetramers provide the most accurate means of quantifying epitope‐specific T cells.

In conclusion, our data have demonstrated that previously characterized bovine CD8 T‐cell responses against the Tp2_49–59_ peptide sequence presented by 2*01201 are composed of non‐cross‐reactive T‐cell subsets specific for Tp2_49–59_/2*01201 and Tp2_50–59_/2*01201. Analysis of TRB genes expressed by responding T cells and structural modelling of the peptide–MHC complexes have shown that these populations express distinct TCR repertoires and that the Tp2_49–59_/2*01201 and Tp2_50–59_/2*01201 pMHC I complexes adopt different surface topographies that account for lack of cross‐reactivity of the respective T‐cell specificities.

## Disclosures

The authors declare no conflicts of interest.
